# Can natural language processing help differentiate inflammatory intestinal diseases in China? Models applying random forest and convolutional neural network approaches

**DOI:** 10.1186/s12911-020-01277-w

**Published:** 2020-09-29

**Authors:** Yuanren Tong, Keming Lu, Yingyun Yang, Ji Li, Yucong Lin, Dong Wu, Aiming Yang, Yue Li, Sheng Yu, Jiaming Qian

**Affiliations:** 1 Department of Gastroenterology, Peking Union Medical College Hospital, Chinese Academy of Medical Sciences and Peking Union Medical College, Beijing, 100730 China; 2 Department of Automation, Tsinghua University, Beijing, 100084 China; 3 Center for Statistical Science, Tsinghua University, Beijing, China, Beijing, 100084 China; 4 Department of Industrial Engineering, Tsinghua University, Beijing, 100084 China; 5 Institute for Data Science, Tsinghua University, Beijing, 100084 China

**Keywords:** Inflammatory bowel disease, Intestinal tuberculosis, Natural language processing

## Abstract

**Background:**

Differentiating between ulcerative colitis (UC), Crohn’s disease (CD) and intestinal tuberculosis (ITB) using endoscopy is challenging. We aimed to realize automatic differential diagnosis among these diseases through machine learning algorithms.

**Methods:**

A total of 6399 consecutive patients (5128 UC, 875 CD and 396 ITB) who had undergone colonoscopy examinations in the Peking Union Medical College Hospital from January 2008 to November 2018 were enrolled. The input was the description of the endoscopic image in the form of free text. Word segmentation and key word filtering were conducted as data preprocessing. Random forest (RF) and convolutional neural network (CNN) approaches were applied to different disease entities. Three two-class classifiers (UC and CD, UC and ITB, and CD and ITB) and a three-class classifier (UC, CD and ITB) were built.

**Results:**

The classifiers built in this research performed well, and the CNN had better performance in general. The RF sensitivities/specificities of UC-CD, UC-ITB, and CD-ITB were 0.89/0.84, 0.83/0.82, and 0.72/0.77, respectively, while the values for the CNN of CD-ITB were 0.90/0.77. The precisions/recalls of UC-CD-ITB when employing RF were 0.97/0.97, 0.65/0.53, and 0.68/0.76, respectively, and when employing the CNN were 0.99/0.97, 0.87/0.83, and 0.52/0.81, respectively.

**Conclusions:**

Classifiers built by RF and CNN approaches had excellent performance when classifying UC with CD or ITB. For the differentiation of CD and ITB, high specificity and sensitivity were achieved as well. Artificial intelligence through machine learning is very promising in helping unexperienced endoscopists differentiate inflammatory intestinal diseases.

**Conference:**

The abstract of this article has won the **first prize of the Young Investigator Award** during the **Asian Pacific Digestive Week (APDW) 2019** held in Kolkata, India.

## Key summary

### Summarize the established knowledge on this subject

● Differential diagnosis among UC, CD and ITB is an important clinical problem.

● Endoscopy is very important in differential diagnosis among UC, CD and ITB.

● Standard terminology system has been built for describing endoscopic images and many endoscopic features are summarized.

● The accuracy of differential diagnosis relies on the experience of clinical doctors.

### What are the significant and/or new findings of this study?

● This study built classifiers with two algorithms that had high accuracy and recall rate, using the descriptions of endoscopic images in the form of free-text.

● The classifiers could allow clinical doctors to make accurate differential diagnosis barely based on objective descriptions of the endoscopic images.

● The classifiers could help find new features or new combination of features that could contribute to differential diagnosis

● This research provided a new point of view of combining natural language processing and clinical endoscopy.

## Introduction

Inflammatory bowel disease (IBD), including ulcerative colitis (UC) and Crohn’s disease (CD), are idiopathic and chronic digestive tract inflammatory diseases with repeated remission and relapses. Intestinal tuberculosis (ITB) is intestinal inflammation due to *Mycobacterium tuberculosis* infection. The ultimate courses and prognoses of IBD and ITB can be different. For example, ITB can be cured with early diagnosis and proper antituberculosis treatment, while recurrences of CD and UC are common and require life-long follow-up. Misdiagnosis and inappropriate treatment can cause either a prolonged disease course or severe adverse effects [1]. Thus, timely and accurate diagnosis and differentiation of IBD and ITB are very important, especially in China, which has the third-highest tuberculosis incidence rate in the world according to the World Health Organization [2].

Endoscopic examination is of vital importance in the diagnosis of IBDs and is always conducted first. The endoscopic features of IBDs and ITB have been well described [3–5]. However, the three inflammatory intestinal diseases, especially ITB and CD, can be difficult to distinguish because they have very similar manifestations in terms of both clinical symptoms and endoscopic appearance. This fact often causes incorrect endoscopic diagnosis and results in delayed treatment.

There have been some studies focused on performing differential diagnosis between IBD and ITB. Yue Li et al. pointed out that interferon γ-release assays could help distinguish CD and ITB [6]. Ren Mao et al. proposed that computed tomographic enterography assisted in differentiating CD and ITB [7]. These studies conducted differentiation from a traditional point of view, which could not provide immediate feedback to clinicians.

Some recent studies used statistical methods to make differentiations. Yao He et al. built a comprehensive diagnostic nomogram to differentiate between CD and ITB [8]. Y. J. Lee et al. formulated a scoring system to evaluate the weight of each endoscopic feature for identifications of ITB and CD [9]. These studies provided novel insights from multiple aspects into differential diagnosis between IBDs and ITB. However, the sample sizes in these studies were relatively small. In addition, the scoring system developed by Y. J. Lee has not been validated in a subsequent group; thus, the result might be overly optimistic. Another point is that previously proposed diagnosis methods require much manual work and can be time consuming; thus, they are not able to provide timely insights for clinical doctors.

The aim of this study was to perform more accurate endoscopic diagnosis and differentiation of IBD and ITB with the help of natural language processing and machine learning in order to assist physicians, especially those with limited experience. In addition, the applications of these novel algorithms to clinical studies would add to the literature, which could promote the application of artificial intelligence to clinical problems.

## Patients/material and methods

### Overview of the study

With standard terminologies for clinical doctors to describe endoscopic images already established [10–12], we developed several classifiers based on different algorithms to automatically classify UC, CD and ITB with free-text endoscopic descriptions as the input.

### Study population

Electronic health records (EHRs) of a total of 6399 consecutive patients who had undergone colonoscopies in Peking Union Medical College Hospital (PUMCH) and were clinically diagnosed as having UC (*n* = 5128), CD (*n* = 875), or ITB (*n* = 396) from January 2008 to November 2018 were collected successively. This research was approved by the Ethics Committee of Peking Union Medical College Hospital on September 31st, 2018 (IRB S-K894).

The clinical diagnoses of UC and CD were made via a combination of medical history, endoscopic features, pathological features, and treatment follow-up based on the Chinese consensus of IBD (2018) by IBD specialists in this hospital. Diagnosis of ITB was obtained by the presence of any of the following: (1) positive acid-fast bacilli on histological examination or positive *M. tuberculosis* culture; (2) radiological, colonoscopic, and/or other proven TB; or (3) full response to anti-TB therapy. Colonoscopies were performed by well-trained endoscopists at PUMCH using Olympus CF-Q260 or H260 colonoscopes.

Based on the well-established terminology used by endoscopists to describe colonoscopic images, we extracted descriptions of colonoscopic images of the patients’ index colonoscopy in the form of free text. Clinically confirmed diagnoses extracted from the hospital information system (HIS) were used as labels.

### Data processing

**Fig. 1:**
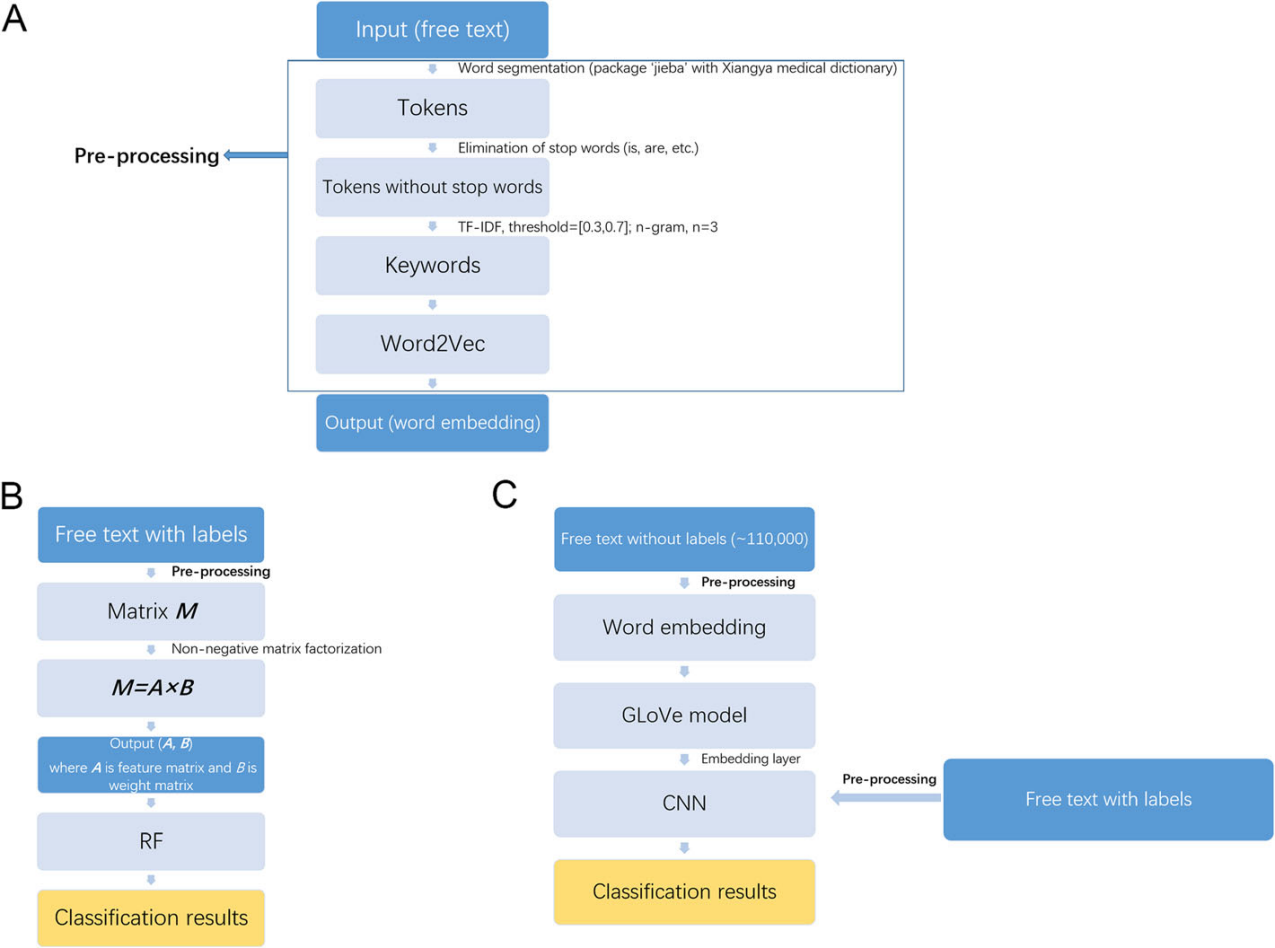
The flow chart of data processing. **a**: the pre-processing which would be used later in RF and CNN; **b**: flow chart of RF; **c**: flow chart of CNN

Figure 1 shows the flow path of data processing. An example of input data could be found in the supplementary material.

The image descriptions were preprocessed with natural language processing (NLP) techniques to extract linguistic features before being input into the classifiers. First, Chinese word segmentation was applied to the description to tokenize the input text, using the Python package ‘jieba’ and enhanced by the Xiangya Professional Medical Dictionary. Punctuation and words without actual clinical meanings such as ‘patients’ and ‘prepare for the examination’ were deleted.

The second step of NLP was keyword filtering, which aimed to identify informative keywords in the description. Term frequency-inverse document frequency (TF-IDF) was applied to filter keywords. The TF-IDF value was defined as follows:

$$ \mathrm{TF}\ \left(\mathrm{X}\right)=\frac{\mathrm{Number}\ \mathrm{of}\ \mathrm{times}\ \mathrm{X}\ \mathrm{appears}\ \mathrm{in}\ \mathrm{the}\ \mathrm{document}}{\mathrm{Total}\ \mathrm{number}\ \mathrm{of}\ \mathrm{terms}\ \mathrm{in}\ \mathrm{the}\ \mathrm{document}}, $$

$$ \mathrm{IDF}\ \left(\mathrm{X}\right)=\mathrm{In}\ \frac{\mathrm{Total}\ \mathrm{number}\ \mathrm{of}\ \mathrm{documents}}{\mathrm{Number}\ \mathrm{of}\ \mathrm{documents}\ \mathrm{containing}\ \mathrm{X}}, $$

$$ \mathrm{TF}\hbox{-} \mathrm{IDF}=\mathrm{TF}\times \mathrm{IDF}. $$

Terms whose TF-IDF values for a document were out of the range of [0.3, 0.7] were removed from the input for that document.

The last step of NLP was dimension reduction with non-negative matrix factorization (NMF) [13], which could improve the interpretability of the extracted features and allow clinical doctors to understand the results better.

In addition to single words, N-grams and L1 regularization (also known as the least absolute shrinkage and selection operator, or LASSO) were applied in the above process as well. Details were provided in Supplementary Materials for conciseness.

### Development of classifiers

Random forest (RF) is appreciated for the advantage in weighting the importance of features. Convolutional neural networks (CNNs) were selected for their capability to extract features automatically. In addition, all algorithms applied are able to analyze free text directly, thus requiring little manual work.

RF was applied to two-class classifications (UC and CD, UC and ITB, and CD and ITB) and the three-class classification of UC, CD and ITB, while the CNN was applied to the three-class classification and the CD/ITB classification. The reason for not applying the CNN to the two-class classifications including UC was an unbalanced sample number.

The labeled dataset was randomly split into a training group (70%) and testing group (30%) for both RF and CNN.

#### Random forest

RF was applied to data processed by NMF. The RF parameters can be found in Supplementary Materials Table 1 Due to the unbalanced data, cost-sensitive learning was employed in order to assign different weights to different diseases. This approach could improve the performance of the model of CD and ITB, which had a small number of samples.

For choosing hyperparameters on RF, ten-fold cross-validation was applied on the training set (70% of total data). The training set were split into ten equal subsets randomly. Nine subsets were used to train for hyperparameters, while the remaining subset was used as the validation set. The chosen hyperparameters were then applied for further train and test.

Regarding feature extraction, we first extracted 50 features sorted by variable importance of RF, which comprised phrases produced by segmentation. All the features were then reviewed by two experienced clinical doctors to combine similar features and omit meaningless or duplicated features.

#### Convolutional neural network

To train CNN models, we extracted approximately 110,000 descriptions from the endoscopic center of PUMCH without labels. The data were employed to train a GloVe [14] model for word embedding, which was used to initiate the CNN embedding layer.

The CNN model applied a structure similar to the Text-CNN model proposed by Yoon Kim [15]. A word list was built, and an integer was allocated to each word. The input sentences were segmented into words and represented by a corresponding integer sequence. The integer sequence was then embedded into a 100-dimension vector. The vectors were used as the input for the CNN. The parameters of the CNN can be found in Supplementary Materials Table 2.

The model was optimized by the Adam algorithm [16]. FocalLoss was used as the loss function to handle the imbalanced sample sizes and accelerate convergence [17]. A total of 100 iterations were employed to allow the model to converge.

### Result visualization

T-distributed stochastic neighbor embedding (t-SNE) [18] was applied to reduce the dimensionality of the features to visualize the results.

### Statistical analysis

The receiver operator characteristic (ROC) [19] curve was applied to evaluate the performance of the two-class classifiers. The sensitivity, specificity and area under the curve (AUC) were calculated. AUC could evaluate the classifiers globally and was not sensitive to the ratio of positive and negative samples. The precision (also known as the positive predictive value), recall (also known as sensitivity), and F1 score were used to evaluate the performance of the three-class classifier. The statistics mentioned above were defined as follows:

$$ \mathrm{Recall}=\frac{\mathrm{True}\ \mathrm{Positives}}{\mathrm{Positives}}=\frac{\mathrm{True}\ \mathrm{Positives}}{\mathrm{Ture}\ \mathrm{Positives}+\mathrm{False}\ \mathrm{Negatives}}, $$

$$ \mathrm{Specificity}=\frac{\mathrm{True}\ \mathrm{Negatives}}{\mathrm{Negatives}}, $$

$$ \mathrm{Precision}=\frac{\mathrm{True}\ \mathrm{Positives}}{\mathrm{True}\ \mathrm{Positives}+\mathrm{False}\ \mathrm{Positives}}, $$

$$ \mathrm{F}1\hbox{-} \mathrm{score}=\frac{2\mathrm{Precision}\times \mathrm{Recall}}{\mathrm{Precision}+\mathrm{Recall}}. $$

All statistical analyses were performed by R 3.5.1 software and Python 3.7.

## Results

### Basic characteristics of the study population

For the 6399 patients enrolled in this study, the male-to-female ratios were 1.12:1, 2.59:1, and 0.79:1 for patients with UC, CD and ITB, respectively. The mean (standard deviation, SD) ages of patients with UC, CD and ITB were 42.94 ± 13.66, 36.04 ± 14.15, and 42.21 ± 16.10, respectively. The basic characteristics of the 6399 patients are summarized in Table 1.

**Table 1:** Basic demographic characteristics of the 6399 patients enrolled in the study

	Ulcerative colitis *N* = 5128	Crohn’s disease *N* = 875	Intestinal tuberculosis *N* = 396
Sex, male(%)	2710 (52.8%)^***^	631 (72.1%)^***^	175 (44.2%)^*^
Age (Mean ± SD), years	42.94 ± 13.66	36.04 ± 14.15	42.21 ± 16.10

*: *P* < 0.05; ***: *P* < 0.001. Null hypothesis: male (%) = 50%

### Convergence of the CNN

After approximately 20 iterations, the model reached convergence, which indicated that the trained CNN model had stabilized. Supplementary Fig. 1 shows the convergence of the loss and the accuracy of the CNN.

### Differential diagnosis between UC and CD

The performance of the classifier is presented in Table 2. The sensitivity, specificity and AUC were 0.890, 0.837 and 0.936, respectively, when using RF. The CNN was not applied. The features extracted by RF to distinguish UC and CD are summarized in Table 3. Mucosal damage characteristics including diffuse congestion, ulcers with purulent secretion, loss of vascular texture, bleeding tendency after touch, and location of lesions for example rectum involved, without lumen stenosis, without involvement of ileocecal valve are extracted features indicating UC.

**Table 2:** Performances of Classifiers

	Sensitivity		Specificity		AUC			
	RF	CNN	RF	CNN	RF	CNN		
UC and CD	0.89	–	0.84	–	0.94	–		
UC and ITB	0.83	–	0.82	–	0.89	–		
CD and ITB	0.72	0.90	0.77	0.77	0.82	0.91		
	Precision		Recall		F1 score		Accuracy	
	RF	CNN	RF	CNN	RF	CNN	RF	CNN
UC	0.97	0.99	0.97	0.97	0.97 ± 0.01	0.98 ± 0.01		
CD	0.65	0.87	0.53	0.83	0.58 ± 0.02	0.85 ± 0.01		
ITB	0.68	0.52	0.76	0.81	0.72 ± 0.02	0.63 ± 0.02		
							0.77 ± 0.02	0.88 ± 0.01

*AUC* Areas under the curve; *UC* Ulcerative colitis, *CD* Crohn’s disease, *ITB* Intestinal tuberculosis, *RF* Random forest, *CNN* Convolutional neural network

The confidence interval of accuracy score is calculated by

$$ accuracy\pm {z}_{0.975}\sqrt{\frac{accuracy\left(1- accuracy\right)}{n}} $$

The confidence interval of F1 score is estimated by the bootstrap method

**Table 3:** Differential features of UC and CD

Features	UC	CD
Mucosal diffuse congestion	+	
Ulcers covered with white exudates		+
Ulcers with purulent secretion	+	
Undamaged haustral pattern		+
Loss of vascular texture	+	
Rectum involved	+	
Spot mucosal erosion		+
Lumen stenosis		+
Bleeding tendency after touch	+	
Involvement of ileocecal valve		+

*UC* Ulcerative colitis, *CD* Crohn’s disease

### Differential diagnosis between UC and ITB

The performance of the classifier is reported in Table 2. The sensitivity, specificity and AUC were 0.833, 0.818 and 0.892, respectively, when using RF. The CNN was not applied. The features extracted by RF are summarized in Table 4. Undamaged terminal ileum mucosa, pseudopolyps or neoplasm, undamaged haustral pattern, and involvement of ileocecal valve are extracted major features indicating ITB.

**Table 4:** Differential features of UC and ITB

Features	UC	ITB
Undamaged terminal ileum mucosa		+
Pseudopolyps		+
Loss of vascular texture	+	
Smooth mucosa		+
Undamaged haustral pattern		+
Ulcers with purulent secretion	+	
Diffuse mucosal congestion	+	
Rectum involved	+	
Involvement of ileocecal valve		+
Bleeding tendency after touch	+	

*UC* ulcerative colitis, *ITB* intestinal tuberculosis

### Differential diagnosis between CD and ITB

The performance of the classifier is reported in Table 2. The sensitivity, specificity and AUC were 0.723, 0.772 and 0.816 when using RF and 0.900, 0.771 and 0.910 when using the CNN, respectively. The features extracted by RF are summarized in Table 5. Involvement of ileocecal valve and undamaged ileum are extracted as top features for differentiating ITB from CD. Features including lumen stenosis, mucosal congestion, loss of vascular texture and ulcers covered with white exudates indicate CD.

**Table 5:** Differential features of CD and ITB

Features	CD	ITB
Loss of vascular texture	.+	
Involvement of ileocecal valve		+
Ulcers covered with white exudates	+	
Pseudopolyps	+	
Lumen stenosis	+	
Mucosal congestion	+	
Undamaged ileum		+

*CD* Crohn’s disease, *ITB* Intestinal tuberculosis

### Visualization of the classification

**Fig. 2:**
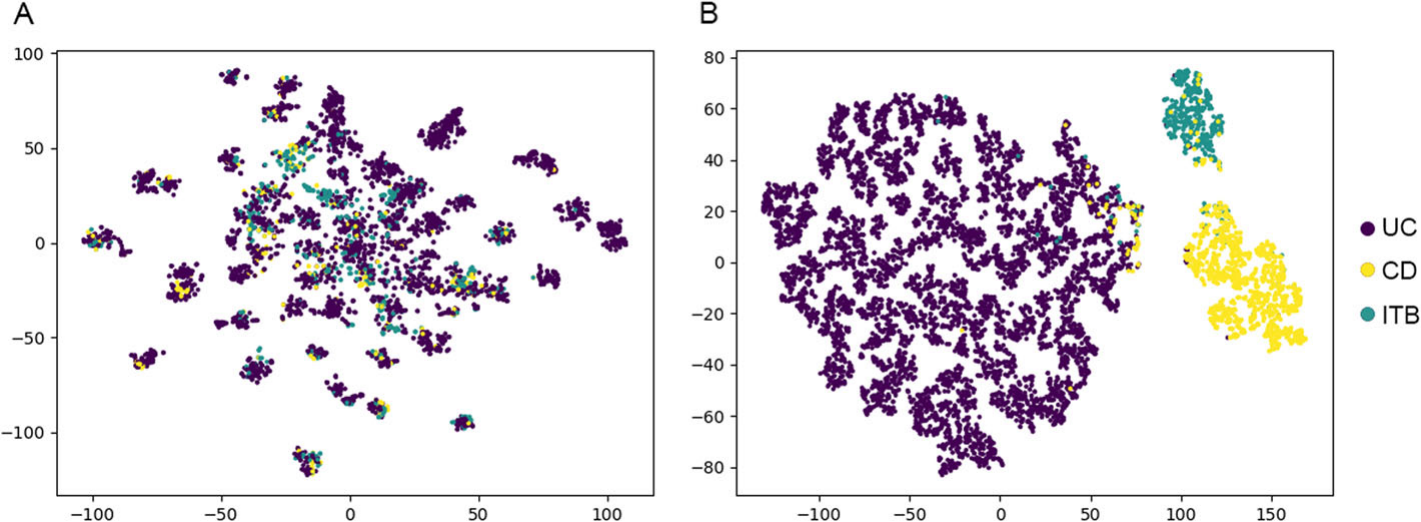
The visualization of the result of two classifiers. **a**: the classifier built by RF; **b**: the classifier built by CNN. The distance between two dots was proportional to the similarities of the corresponding endoscopic descriptions. Different color of the dots represented different diseases. Purple: Ulcerative colitis (UC); yellow: Crohn’s disease (CD); green, Intestinal tuberculosis (ITB)

To present the classification results more intuitively, the results are visualized in Fig. 2 by the t-SNE algorithm. Note that the X and Y axes are the result of dimension reduction of features and bear no real world meaning. Each dot represents an endoscopic description of a patient. The distance between two dots is proportional to the dissimilarity of the extracted features of two samples. The CNN almost grouped all same diseases together, while RF formed many small clusters and many of which had mixed colors. This shows that the CNN could automatically extract features that were highly capable at characterizing the three differential diagnoses. Further research on interpreting the feature representations from CNN is warranted.

## Discussion

To the best of our knowledge, this is the first study to apply RF and the CNN to realize automatic classification of IBD and ITB based on endoscopic results in the form of free text. Classifiers built by both RF and the CNN had extremely high sensitivity and specificity for two-class classification problems UC and CD and UC and ITB and the three-class classification problem (UC, CD and ITB), which indicates that UC can be potentially automatically diagnosed based on a simple objective description, while differential diagnosis of CD and ITB can be strongly indicated by the classifier.

Diffuse mucosal damage, bleeding tendency and ulcer with purulent secretion were major features that led to a classification of UC. Location of the lesion was also an indicator. However, continuous lesions, which were always mentioned in diagnoses in other studies, were not clearly proposed. The reason could be that mucosal diffuse damage covers the meaning of continuous lesions; thus, continuous lesions were not considered an independent feature. In general, the features automatically extracted by RF were similar to those reported in previous studies and guidelines [3–5].

Differential diagnosis between CD and ITB has always been clinically challenging. A study showed that approximately 65% of patients with CD had been incorrectly diagnosed as having ITB at least once in China [9]. Another study found that approximately 40% of CD patients had received a trial anti-TB treatment because of misdiagnosis [20]. Although histologic or pathologic features including caseating granuloma, isolation of *M. tuberculosis* or a positive acid-fasting staining result can provide extremely strong evidence for TB diagnosis, previous studies pointed out that these examinations were positive in fewer than 50% patients [9].

Studies focused on endoscopic differential diagnosis have raised several differential diagnosis points, including the number of involved colon segments, ileocecal valves, direction of ulcers, special appearance (cobblestone, scars and pseudopolyps) and location of lesions [1]. However, the weights and potential combinations of these points have not been clarified. In addition, these clinical features for differential diagnosis often exist in an individual patient simultaneously. The two reasons above can make doctors, especially those with limited experience, have great difficulty in making an accurate endoscopic diagnosis. The classifiers built in this research address this gap by assigning weights to each feature, allowing doctors to simply record the endoscopic image faithfully and objectively and could result in high precision and a high recall rate, which would benefit clinical work.

In addition, the classifiers built by RF found some features that were new or rarely noted before. Ulcers covered with white exudate were considered a sign of CD instead of ITB. The white exudate could be due to an inflammatory response. Although we cannot claim the diagnostic value of this point immediately, we hope that this finding can lead to further research. Mucosal congestion and loss of vascular textures used to be considered features of UC. However, the classifier considered these features signs of CD when performing differential diagnosis between CD and ITB, which might provide some new insights. Other features extracted by RF are reported in Table 5; these were mentioned in previous studies or guidelines [3–5].

The detailed features of ulcers (longitudinal, transverse, cobblestone appearance, etc.) were not considered important features for differential diagnosis of CD and ITB by the classifier in this study. This is different from the findings of previous studies. We suppose that the reason may be that the patients with typical features (e.g., cobblestone appearance of CD) comprised only a very small portion of all patients enrolled in the research. These signs might be important, but the statistical power is not strong enough to detect this difference. In other words, examination of these features has high specificity but low sensitivity. Thus, these features are not proper for differential diagnosis of all patients.

From the points above, features extracted by RF could also provide a new view for doctors to make differential diagnosis. Classifiers built by RF could find and assign different weights to each feature. Besides, these classifiers could also combine the features together to provide potential extra information. The two points above might help doctors to make better differential diagnosis. In addition, Interpreting the RF results in comprehensive manner may help encourage the doctors and clinicians to use machine learning tools in healthcare field.

The CNN could not be applied to the two-class classifiers including UC in this study because of extremely unbalanced sample sizes (UC: CD = 5128:875; UC: ITB = 5128:396), although CNNs often perform better in classification problems. This phenomenon is caused by the different morbidity associated with different types of inflammatory intestinal diseases and is a consequence of consecutive enrollment of patients. On the other hand, the CNN performed better than RF in the classification of CD and ITB. However, the weakness of the CNN was its interpretability, which meant that we could not figure out the classification features used by the CNN. Therefore, from the perspective of a physician, the result given by the CNN can be a reference but without supporting details; thus, it is difficult to accept the outcome of this approach as the final clinical diagnosis. However, further clinical trials might provide further evidence, and the CNN model could be used to assist physicians. Large-cohort studies may help improve the credibility of the CNN as well.

Regarding the three-class classification problem, RF could also extract some features. However, the attribution of each feature could be difficult to figure out because a single ‘either-or’ feature such as damaged/undamaged mucosa was not sufficient for three-class classification. Linear combinations of extracted features might be used with variable coefficients. The complexity of the three-class classifier might have caused its low performance. Although the CNN performed better than RF in the three-class classification problem according to Table 2 and Fig. 2, the interpretability of the CNN will always be a barrier that is difficult to remove. However, the precision and recall rate of UC were extremely high, even when using the three-class classifier built by RF; thus, only patients who are suspected of having CD or ITB would need further classification. The two-class classifier of CD and ITB discussed above could then be applied.

Due to the similarity of the endoscopic image of CD and ITB, some cases might could not be differentiated purely by endoscope and needed further examinations. The upper limit value of the proportion that could be differentiated of ITB and CD depended on both experience of doctors and the power of endoscopic diagnosis criteria. The classifiers built in this research could help unexperienced doctors to make more accuracy diagnosis while the RF classifiers could find some new features for diagnosis, thus might potentially improve the power of diagnosis criteria.

There are several limitations of this study. First, Behcet’s disease was not taken into consideration because it is often considered a systemic disease and with variable initial involvement, including genital aphthae, gastrointestinal involvement, skin lesions, vascular disease, neurologic disease, and arthritis [20], and it was not appropriate for inclusion in this study. Second, although we assumed the existence of a complete terminology, subjectivity might still cause bias when endoscopists write the descriptions. In addition, endoscopy doctors were not completely blind to other clinical examination results, which could cause subjectivity bias. We have used the first endoscopic description, which had least additional information, to minimize these bias. However, to eliminate these bias, classification algorithms directly based on endoscopic images might be preferred, which requires much more computational resource. We are looking forward to further researches that cooperated by clinical doctors and technicians. Third, the numbers of patients with UC, CD and ITB involved in this research were not balanced (5128 UC, 875 CD and 396 ITB). This was mainly because of the different prevalences and morbidities of these three diseases. A study with nonconsecutive patients might have a more balanced sample but would have potential selection bias. Lastly, these research was conducted in Chinese. However, the NLP models could be easily applied on other languages.

## Conclusion

This was the first study that applied RF and a CNN to realize the automatic classification of known inflammatory intestinal diseases. The performance of the classifiers was reasonable. In addition, RF found some new features that were potentially valuable for further research. Artificial intelligence through machine learning is very promising in helping unexperienced endoscopists differentiate inflammatory intestinal diseases.

## Supplementary information

**Additional file 1: Supplementary Table 1**. Parameters applied in the RF model. **Supplementary Table 2**. Parameters applied in the CNN model. **Supplementary Figure. 1**. The convergence of loss and accuracy of CNN.

## Data Availability

The datasets of involved patients’ endoscopic descriptions analyzed during the current study are not publicly available due to patient privacy and the requirement of the Ethics Committee of Peking Union Medical College Hospital. If there are any need, please contact the corresponding authors.
