# Supplementary material for: Can natural language processing help differentiate inflammatory intestinal diseases in China? Models applying random forest and convolutional neural network approaches
Source: BMC Med Inform Decis Mak. 2020 Sep 29;20:248. doi: 10.1186/s12911-020-01277-w (PMC7526202; doi:10.1186/s12911-020-01277-w)
Supplement: Supplementary file 1 — Additional file 1: Supplementary Table 1. Parameters applied in the RF model. Supplementary Table 2. Parameters applied in the CNN model. Supplementary Figure. 1. The convergence of loss and accuracy of CNN. [file 12911_2020_1277_MOESM1_ESM.docx]

Supplementary table 1. Parameters applied in the RF model.

| Parameters | Values |
| --- | --- |
| Number of estimators | 200 |
| Maximum depth | 13 |
| Maximum features | Square root of sample size |
| Minimum samples of a leaf | 20 |
| Minimum samples for splitting a leaf | 80 |

Supplementary table 2. Parameters applied in the CNN model.

| Embedding layer | 100-dimension vectors |
| --- | --- |
| Convolution layer | 64 convolutional kernels with window width 2-,4, height 100. |
| Maxpooling layer | 1-Maxpooling |
| Linear layer | Dimension=50×3 |
| Dropout layer | Dropout rate=0.5 |

Supplementary Figure 1. The convergence of loss and accuracy of CNN. The algorithm converged after 20 iterations.


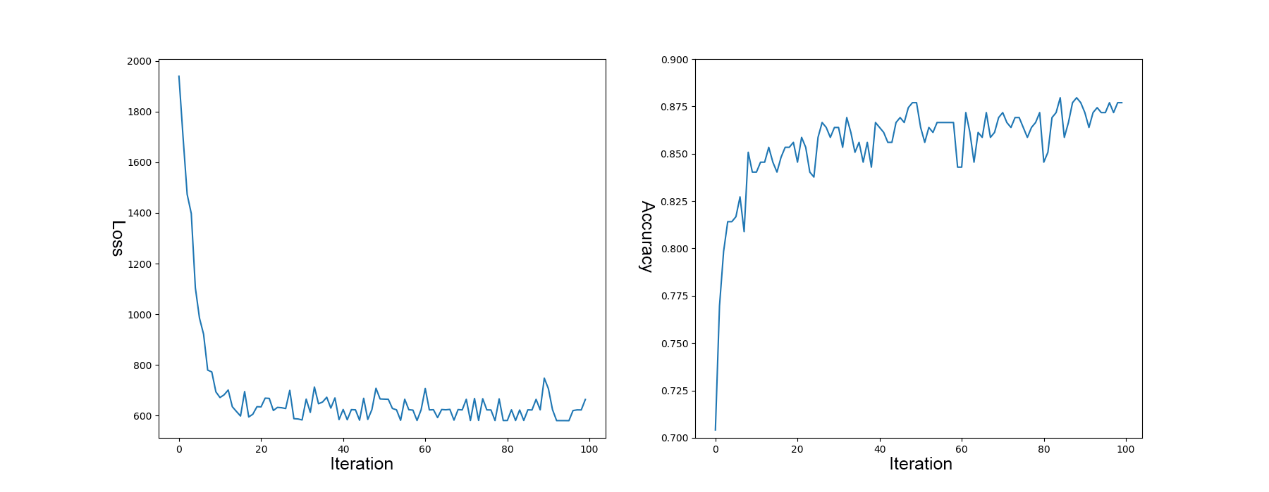


An example of input data

克罗恩病

肠道准备可，观察基本满意。钩拉法循腔进镜至回肠末段10cm，所见小肠黏膜大致正常。回盲瓣呈唇形，回盲部、横结肠肝曲、脾曲及降结肠见多发不规则溃疡，呈纵行或圆形，降结肠溃疡占据3/4管腔，表面覆薄白苔，底部结节样不平，肠腔略窄，内镜尚能通过，周边黏膜肿胀隆起，并有多发炎性息肉形成，可见黏膜疤痕；直肠粘膜光整，未见糜烂、溃疡及异常隆起。

Label: Crohn’s disease (CD)

Bowel preparation and observation is basically satisfactory. The endoscope entered around 10cm into the end of the ileum by using hook-pull method. The small intestinal mucosa was generally normal. The ileocecal valve was lip-shaped, and multiple irregular ulcers were observed in ileocecal area, the liver flexure of the transverse colon, the spleen flexure and descending colon. The ulcers were longitudinal or round. The descending colon ulcer occupied three quarters of the lumen, covered with thin white coating on the surface, and nodules at the bottom. The intestinal cavity was slightly narrow, the endoscope can still pass. The surrounding mucosa was swollen, and multiple inflammatory polyps were formed. Mucosal scars are visible. The rectal mucosa was smooth, no erosions, ulcers or abnormal eminence were observed.
